# Long-Term Biobanking of Intact Tissue from Lipoaspirate

**DOI:** 10.3390/jcm8030327

**Published:** 2019-03-08

**Authors:** Michael S. Badowski, Angela Muise, David T. Harris

**Affiliations:** AHSC Biorepository, 1501 N. Campbell Ave., AHSC 6122, PO box 245221, The University of Arizona, Tucson, AZ 85724, USA; muisea@email.arizona.edu (A.M.); davidh@email.arizona.edu (D.T.H.)

**Keywords:** adipose tissue, cryopreservation, autologous, fat grafting, cryogenic storage, lipoaspirate, cosmetic surgery, regenerative medicine

## Abstract

Autologous fat grafting has now been extensively and successfully performed for more than two decades. Although most adipose grafts and adipose-derived MSC therapies are done with fresh tissue, cryopreservation of tissue allows for much greater flexibility of use. Over the course of five years, 194 cryopreserved adipose samples were thawed and then returned to the collecting physician for subsequent autologous applications. Samples were stored with a mean cryogenic storage time of 9.5 months, with some samples being stored as long as 44 months. The volumes of tissue stored varied from 12 cc to as large as 960 cc. Upon thawing, the volume of recovered whole adipose tissue averaged 67% of the original amount stored for all samples, while the samples that were stored for longer than one year averaged 71%. Recovery was not found to be a function of length of time in cryopreservation. No significant relationship was found between tissue recovery and patient age. While an average recovery of 67% of volume frozen indicates that the use of banked and thawed tissue requires a larger amount of sample to be taken from the patient initially, an experienced clinician easily accomplishes this requirement. As cryopreservation of adipose tissue becomes more commonplace, physicians will find it helpful to know the amount and quality of tissue that will be available after thawing procedures.

## 1. Introduction

Uses for autologous fat from lipoaspirate have greatly increased over the years. Autologous fat grafting is now commonplace for reconstructive, cosmetic, and regenerative medicine applications [1,2,3]. A variety of cellular and other therapies can include use of whole lipoaspirate, enzymatically derived stromal vascular fraction (SVF), mechanically derived microfat, or cultured and expanded adipose derived MSCs. While these and other uses become more and more standard, they still suffer some inefficiencies due to the need for fresh adipose harvests. Patients that require more than one treatment over timeframes longer than one day often need a separate harvest of lipoaspirate material for these multi-faceted interventions. One method to alleviate this issue is the simple cryopreservation of adipose tissue. The storage of adipose tissue via cryopreservation allows for the physician to dip into a store of therapeutically useful material at will. Without the need for additional adipose harvests the patient enjoys reduced morbidity, discomfort, and cost, while the treating physician has more flexibility over the course of treatments and potentially greater overall efficiency of clinical operations. 

Adipose tissue has been used extensively and successfully for decades. First described late in the 19th century by Neuber [4], it was only minimally used throughout most of the 20th century. This lack of widespread adoption may have been due to poor performance, as “blocks” of adipose tissue were usually transferred with little regard for the required vascularization [5]. Only with the advent of liposuction in the 1980s [6,7], and Coleman’s work in the 1990s [8], did our current concept of adipose tissue transfer emerge. The increases that were seen after Coleman’s systemization of the techniques in 1995 are such that the grafting technique is now commonplace. In the intervening twenty-plus years, a greater understanding of adipose grafting has come from improved harvest techniques, increased knowledge of tissue handling, and a more experienced group of clinicians. In addition to clinical use of freshly harvested tissue, better cryopreservation protocols [9,10,11] and greater experience with post-thaw uses have made an equally strong case for cryopreserved adipose graft utility. Although most adipose grafts are still done with fresh tissue, the cryopreservation of tissue allows for much greater flexibility of use. A large variety of applications are now common in cosmetic uses [12,13] and stem cell therapies [14,15,16]. Many additional experimental applications show great promise—from cardiac repair [17,18] to endocrine therapies [19,20]. More applications are likely forthcoming as the cryopreservation of adipose tissue becomes more commonplace. As these new applications are being developed and refined, it will be important for each of these new therapies to ensure that there is enough raw lipoaspirate to use. Whatever the therapy, the amount of available lipoaspirate will affect the number of cells that are derived from the tissue, and necessarily the potential cell dosage available to the eventual patient. Therefore, the scope of this paper largely deals with raw lipoaspirate and its availability after cryopreservation, as it will be a factor in these therapies.

One advantage of adipose tissue is that the physician can use whatever amount is needed for treatment. Small-scale cosmetic applications include treatment for facial rejuvenation [12,21], fine lines and wrinkles on face and hands [22,23], and scar repair [24,25]. Larger volumes are needed for applications, such as breast reconstruction or enhancement [25,26] or other large area sculpting/remodeling. The amount of tissue needed for different applications can be exceedingly variable. While several hundred milliliters might be used for larger defects or for reconstruction, some uses will require far less. It therefore behooves the clinician to know how much fat would be a useful amount when it is time for the tissue to be applied. Many practitioners presently harvest adipose tissue and use it for reinjection during the same office visit [27]. However, it has been clear for some time that the cryopreservation of adipose tissue for future use is not only a viable option, in many ways it is advantageous. The adipose tissue that was collected from a single harvest can be cryopreserved into multiple aliquots of varied sizes. This approach reduces not only cost to the patient, but also patient discomfort, morbidity, and possible complications. As a general principle, cells that are younger are more healthy, hardy, and generally more useful. As patients age, it would be beneficial for physicians to have a bank of tissue from which they could draw. If these frozen tissues are adipose tissue aliquots from the same patient (only younger), so much the better [28]. As adipose tissue is a rich source of mesenchymal stromal cells (MSCs) and other cells future applications in cellular and regenerative medicine are amendable. As such, this work is meant to act a precursor to more advanced regenerative medicine research. Since the full utilization of freshly harvested tissue is not always possible, the amount that is available at a later date is likely to be an important factor in planning future therapies and treatments.

Cryopreservation of a large amount of tissue allows the medical professional to sample a large store of tissue whenever additional adipose tissue is needed [11]. This approach obviates the need for additional patient harvests with their associated cost and trauma. This may be extremely useful for those patients that may have less than optimal healing. Diabetics, the elderly, rheumatoid arthritics, or others with chronic wounds and ulcerated conditions may benefit the most from this approach as multiple treatments may be indicated. To aid the clinician in determining the amount of adipose tissue that should be harvested for cryopreservation, this study analyzes the recovery yields supporting the utility of cryopreserved adipose tissue for future clinical use.

## 2. Materials and Methods

### 2.1. Tissue Collection and Processing

Lipoaspirate samples were collected from male and female adult patients undergoing voluntary liposuction. All of the samples were obtained with written consent from the donors according to the instructions from the local institution review board at the University of Arizona. No paid compensation was given to subjects. Tissue was collected into sterile vessels and then allowed to separate by sedimentation. In this manner blood, tumescent fluid and oils were removed from tissue. The separated tissue was packaged into syringes and then shipped via overnight or local courier. Upon receipt at the processing laboratory, syringes were placed upright at room temperature for a further round of sedimentation. Any remaining fluids were removed from each syringe along with a small amount of adipose tissue into a sterile tube. The samples were pooled into a single sterile tube when multiple syringes arrived for processing. This extract was used for viability and sterility testing. If no tumescent fluid were present in the packaged syringes, a small amount of adipose tissue was removed and then vigorously mixed with sterile PBS to facilitate testing. 

### 2.2. Sterility Testing

Sample liquids were streaked with a 10 µL sterile loop onto growth plates. Using a sterile loop, the sample was streaked onto MacConkey agar, Sabaroud-dextrose agar, and 5% sheep’s blood tryptone soy agar plates (University of Arizona Bio5 Media Facility, Tucson, AZ, USA). All of the agar plates were incubated and monitored for seven days in a 37 °C dry incubator. The appearance of colonies at any point during the seven days was scored as microbial growth present. In lieu of testing on plates, some of the samples were tested with the BacT/ALERT system (Biomerieux, Marcy l’Etoile, France). For these assays, sample liquid was loaded into a sterile syringe with needle and injected into the culture liquid through a rubber septum. Both the aerobic and anaerobic media bottles were kept for seven days at 37 °C. Any growth within seven days was scored as positive. The sterility results were reported to the physician but are not part of this study.

### 2.3. Histology

Small pieces of adipose tissue were placed on a microscope slide. A second slide was used to smear the tissue across the slide. It was then fixed in methanol for 30 s and placed in 0.1% Crystal Violet for at least one hour. When stain had permeated completely though tissue, the slide was rinsed gently by dipping in clean water until all excess stain was removed. Tissue preparation was allowed to air dry. A cover slip was placed over stained tissue, secured in place with Histomount, and then allowed to dry for a least a day. The slides were viewed on a standard brightfield light microscope.

### 2.4. Cryostorage

Adipose tissue was treated, as previously described [11]. Tissue was loaded into sterile cryostorage bags (Origen Biomedical, Austin, TX, USA) and cooled to 4 °C. Ice cold cryoprotectant was added to achieve a final concentration of 5% DMSO, 1% dextran-40 (Protide Pharmaceuticals, Lake Zurich, IL, USA), and 1% human serum albumen (Octapharma, Lachen, Switzerland). After mixing, the bags were sealed into secondary sterile overwrap. Bags were frozen according to a preprogrammed cooling curve in a controlled rate freezer (Custom Biogenic Systems, Romeo, MI, USA) and then stored in vapor phase LN2 dewar at −180 °C.

### 2.5. Thawing of Frozen Adipose Tissue

Cryostorage bags were rapidly thawed in a 37 °C water bath and washed twice with cold lactated Ringer’s solution. Wash liquid was separated from tissue and the volume of tissue measured in the same manner as samples prior to cryopreservation. Washed tissue was then transferred to 20 cc or 60 cc packaging syringes. Tissue was loaded in a 1:1 ratio, with transport buffer consisting of lactated Ringer’s solution with 1% human serum albumen. Tissue was kept refrigerated in a temperature-controlled shipping container overnight or local courier delivery to the administering clinician. 

## 3. Results

### Adipose Tissue Recovery after Prolonged Cryopreservation and Thawing

**Figure 1:**
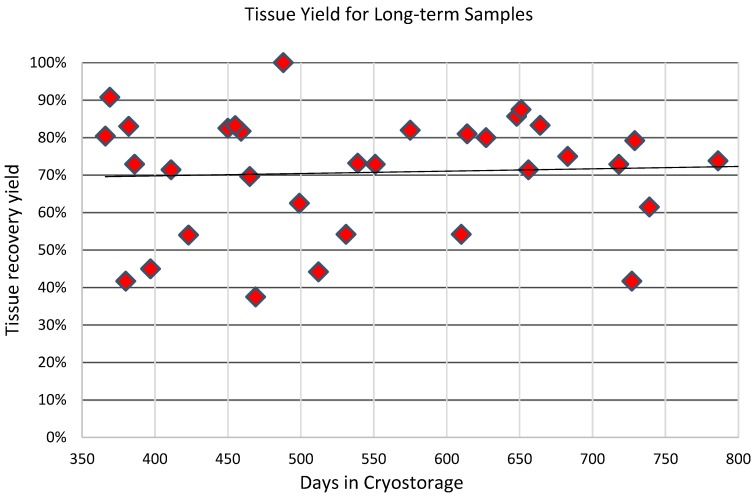
Long-term yield. Adipose tissue was stored for 38 patients for times longer than one year. Upon thaw an average of 71% of original tissue volume was available for subsequent use.

Samples, or aliquots of samples, were in cryostorage for an average approximately 9 ½ months. A total of 181 samples were thawed after various lengths of time following cryopreservation. The samples had been stored for periods ranging from 13 days to more than 44 months. Long-term samples were considered to be in storage for more than one year. 38 samples met this long-term criterion. After the thaw procedures were complete, the amount of tissue recovered was compared to the amount of tissue originally cryopreserved in each sample or aliquot. Due to original removal of waste liquids (blood, tumescent fluid, oil) prior to cryostorage, those volumes were not included in the amount of tissue stored. The average recovery yield of cryopreserved tissue volume was 67% of the original stored amount. Tissue recovery ranged from a low of 21% to a high of 100%. The large majority of samples fell between 50% and 80%. For samples that were classified as long-term, i.e. those in cryostorage for longer than one year, the yields are slightly higher. An average of 71% of tissue volume was recovered after thaw procedures. Figure 1 shows the distribution of recovery yield for these long-term samples. There appears to be no correlation of tissue recovery with length of time in cryostorage.

**Figure 2:**
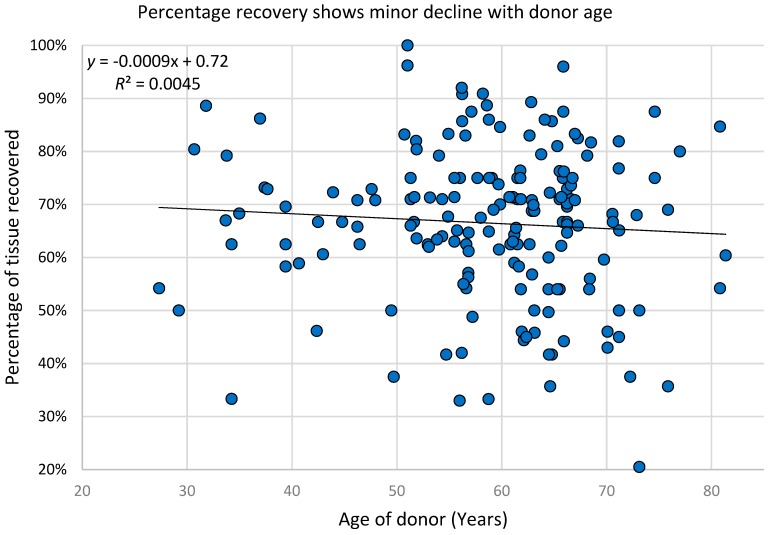
Percentage recovery shows little decline with age. The age of adipose tissue donors ranged from 27 years to 83 years. Regardless of donor age, there was no significant decrease in thawed tissue yield after cryopreservation.

Although the majority of patients in this study ranged from 50 to 70 years of age, the entire patient population was from 27 to 81 years old. The yield of adipose tissue recovery after thawing appears to have very little dependence on the age of the patient. Linear regression analysis shows that, for each additional decade of age, the reduction in recovery is less than one percentage point. Figure 2 shows the distribution of post-thaw tissue yield and its relationship to donor age.

**Figure 3:**
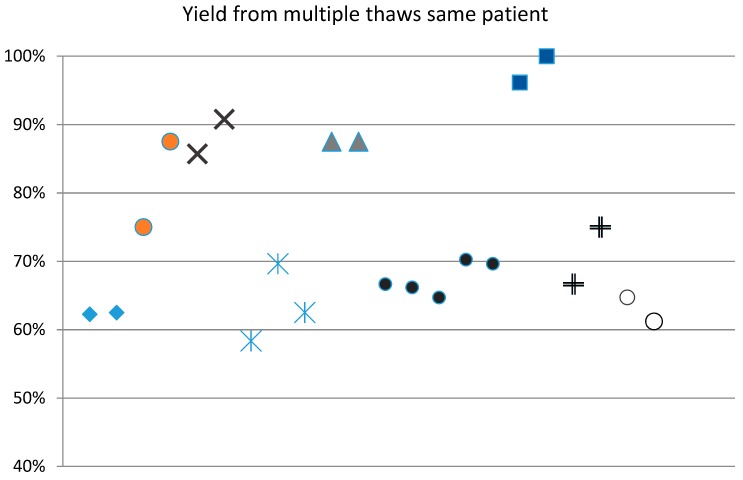
Multiple thaws from same patient are similar. Large adipose samples were stored in multiple smaller aliquots. Individual patients (*n* = 9) are shown here as markers of the same style. Tissue yield from thaws procedures on different days show similar recovery levels.

The adipose samples that were cryopreserved in this study ranged from 12 cc to 960 cc (mean = 242 cc) of the total sample. This volume included some waste materials, such as blood, oils, and tumescent fluid, which were packaged along with the adipose tissue into the collection syringes. These waste fractions were discarded prior to adipose cryopreservation. Samples that were smaller than 12 cc were excluded, but there was no upper limit on the collected volume. Therefore, the amount of lipoaspirate cryopreserved was largely dependent upon how much the physician harvested. Since large samples were stored in multiple aliquots, the sample was, thereafter, available for multiple autologous uses. Some physicians had taken out aliquots of sample on multiple occasions for multiple post-thaw usages. Figure 3 shows the recovery percentages varied less within a set of aliquots from the same donor. 

**Figure 4:**
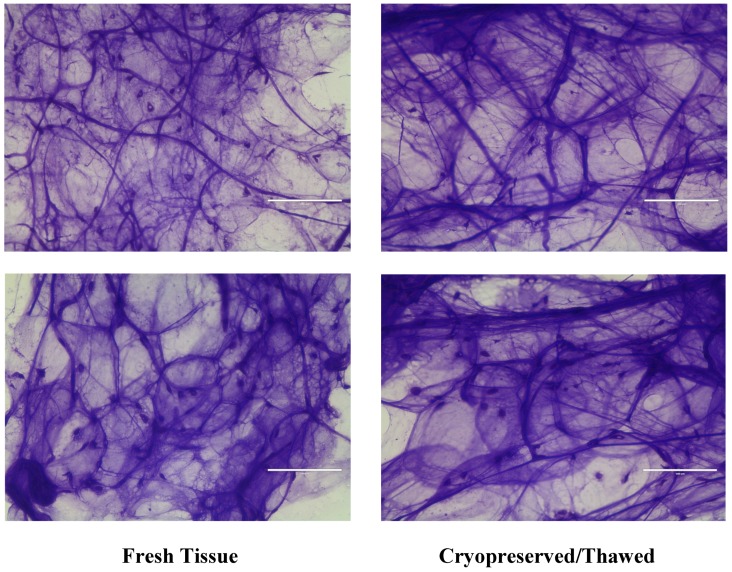
No gross differences between fresh and frozen histology. Adipose tissue was mounted on slides and then stained with crystal violet. No differences in structure are observed in fresh or cryopreserved/thawed tissue.

Tissue prepared onto thick sections and stained with crystal violet was viewed on a light microscope. Samples from fresh adipose tissue and cryopreserved post-thaw adipose tissue were essentially the same. While the large-scale structure of the adipose tissue is disrupted during lipo-extraction, much of the same structures are observed. Intact pieces of tissue, outlines of intact adipocytes, collagen strands and matrix, and density of nuclei can be seen. There appears to be no gross differences in slides that were prepared from fresh or post-thawed adipose tissue. Figure 4 shows two examples of each fresh tissue and post-thawed tissue preparations. It displays that the prepared tissue essentially presents the same histology characteristics from both freshly harvested and cryopreserved/thawed tissue. While the authors understand that H&E staining is the norm for basic histology, crystal violet (CV) was chosen instead of H&E staining for superior visualization of tissue under our conditions. CV has the property of strongly staining both nuclei and intercellular matrix, which is not as robustly seen in H&E. Intracellular structure is more prominently stained with H&E in most cells. However, intracellularly adipocytes are nearly completely all fat filled vacuoles. A characteristic of this feature is that it does not stain with H&E. A strong outline of the shape of the cells can be seen more easily with CV than H&E. These factors led us to choose CV over H&E staining.

## 4. Discussion/Conclusions

Adipose tissue has been shown to have useful for a great many conditions and it has promise for a great many more. It is easy to harvest, easy to handle, and, for many patients, rarely is it in shortage. It provides a rich source of adipose derived MSCs, pericytes, endothelial cells, pre-adipocytes, and others, which are known to have large numbers of current and potential therapies. All else being equal, younger cells are better than older cells [28,29,30]. Therefore, it would seem beneficial for patients to have a bank of stored autologous adipose tissue taken while the patient is young. From this cryopreserved collection, the aliquots can be taken out periodically for the cosmetic needs, regenerative medicine, cellular therapies, and reconstructive applications, which the patient may require in the future. 

In this study, we have seen that the average yield of tissue recovery after thawing is 67% for all samples (21% to 100%). For long-term samples we observed that the average tissue yield recovery is 71%. While slightly higher than the 67% that was found in all samples, it was not found to be significantly higher. The practical application is that, after the thawed tissue is washed and recovered, it allows the physician to easily predict the amount of adipose tissue available for use. A conservative approach allows for a physician to estimate that one-half to two-thirds of the original banked amount will be available for fat transfer applications. Physicians can make this useful estimate for all additional tissue frozen, regardless of aliquot size, overall harvest volume, or length of time in storage. Cryopreservation of cells has been commonplace for decades. Indeed, the samples of adipose tissue act similarly to other properly cryopreserved cells. Essentially, they can be stored indefinitely. The samples remained cooled at liquid nitrogen temperatures of approximately −185 °C. At this temperature essentially, all biological activity is stopped. Without this complete cessation we may expect to see degradation over time. However, properly stored under appropriate conditions, no reduction of recovery yield is seen as the time in cryostorage increases – even over a time frame of years.

An average recovery of 71% for long-term samples of frozen adipose tissue indicates that the use of banked and thawed tissue initially requires a larger amount of sample be taken from the patient. Any clinician that is competent in harvesting technique easily accomplishes this requirement. Our samples in this study were stored in cryopreservation bags that allowed storage of aliquots of adipose tissue of 24 cc or 56 cc per bag. Some physicians had stated that a portion of the fresh lipoaspirate had been used that same day and the remainder had been sent to the processing lab for cryopreservation. However, data regarding the number of these cases and the amount of tissue for each purpose is not available. Outside of this study, we have stored adipose tissue in other types and sizes of vessels that resulted in aliquots of tissue, ranging from 2 cc to more than 200 cc. A wide variety of cryostorage bag sizes are available from our supplier (Origen Biomedical, Austin, TX, USA), as well as other suppliers. This allows for the adipose tissue to essentially be stored in any volume. It seems reasonable that large volumes of tissue are best cryopreserved in multiple aliquots. Multiple aliquots allow the clinician to just thaw the volume that is needed for the present application, while holding the remainder in reserve. While it is possible to refreeze adipose tissue and still utilize it upon a second (or third) thaw, there is an observed reduction in both cell viability and overall volume yield [31]. Once a clinician has an approximate idea of the amount of tissue that is required for a given procedure, the amount of tissue aliquots to be thawed can be predicted. In this way, the clinician is assured of not running out of tissue or of thawing too many bags that will go to waste.

As the adipose tissue is processed and cryopreserved, a small amount of tissue is lost through the normal manipulations that are required. For example, tissue that cannot be easily removed from a syringe or an amount of tissue may become stuck in tubing is unrecoverable. The amount that is lost in the unloading of the collection syringes and the transfer to cryostorage bags is minimal. However, some adipocytes burst and are destroyed during the various processes involved. Moving fragments of lipoaspirate through a small orifice, exposing cells to pressure differences and sheer stresses, changes in osmotic pressure with the cells during cryopreservation and thaw, washing, and centrifugation can all have an effect on the tissue. Taken together, these processes affect a change in the overall volume of the recovered sample. It was noticed that, most often, amounts of tissue were unrecoverable during the thaw and wash procedures and the subsequent transfer of tissue to packaging syringes for transport. Ensuring that the size of cryostorage bag was not overly large as compared to the volume of tissue stored minimized this loss. It was noticed early on that lowest yields were often from cryostorage bags that were filled to less than capacity with adipose tissue. Tissue can be stored in any amount and cryostorage bags come in many sizes. However, more tissue was unrecoverable or lost when the bag was too large for the tissue stored. There was no significant correlation between the total amount of tissue stored and the percentage yield of individual samples. 

There are different reasons for storage of adipose tissue; e.g., for the near-term use for cosmetic purposes. Many of our current tissue retrievals have been for reinjection into the face or hands. Other observed uses have been for larger scale remodeling/reconstruction, such as breast or buttocks. Another reason for the storage of tissue is that adipose tissue is abundant with MSCs, pericytes, and other cells. This potent tissue will, no doubt, be even more useful in the future as yet unknown, regenerative medicine applications are discovered. It is also known that, the younger the stem cells are, the more potent and more useful they will be [16,28,29,30]. Therefore, it behooves patients to collect a sample of adipose tissue and store it at a time as early as is feasible and to keep it stored instead of for short-term use for cosmetic applications. 

Some cellular therapies will depend upon the extraction and expansion of MSCs. For this, small amounts of tissue can be expanded to great use, provided that the viability and cellularity are acceptable. Our previous work has shown this method of cryopreservation and thaw yields viable cell counts that are appropriate for expansion and therefore potential therapies that depend on it. [11] However, in cosmetic and reconstructive cases, the amount and volume of tissue that a physician has to work with is of more importance. As the tissue was processed for thaw (washings, transfers, etc.), only the actual amount of tissue that ended up in the physician’s hands was measured as the final product. As the cryopreservation of adipose tissue becomes more commonplace, physicians will find it helpful to know what will be the likely amount of tissue that will be available after the thaw procedures. The final product ready to use was sufficiently washed and the tissue yield was measured. As the tissue is packaged for use into syringes with osmotically balanced buffer, the physician can immediately use the tissue upon arrival by merely decanting the buffer.

This process is technically very simple for the physician. However, some may not choose such procedures as cryopreservation and fat graft for the concern that it runs afoul of rules and guidance from the U.S. Food and Drug Administration (FDA). It merits some attention that the FDA has produced guidance regarding “processing” of Human Cell, Tissues, and Cellular and Tissue-based Products (HCT/Ps). However, what is herein described falls under CFR Title 21, part 1271.15 (b), which states that ”You are not required to comply with the requirements of this part if you are an establishment that removes HCT/P’s from an individual and implants such HCT/P’s into the same individual during the same surgical procedure”. This exception was further clarified later in FDA Guidance in November 2017. The example of autologous adipose tissue was specifically given and the FDA states “the adipose tissue retains its original form as a connective tissue composed of clusters of adipocytes and other cells surrounded by a reticular fiber network and interspersed small blood vessels. It is then re-implanted into the same patient from whom it was removed in order to achieve the intended effect. We generally would consider the establishment removing and implanting this HCT/P from adipose tissue to qualify for the exception under 21 CFR 1271.15(b)” [32].

The amount of adipose tissue that can be harvested from any given patient is dependent upon the amount of fat on the patient. However, more adipose tissue can nearly always be taken than what usually is taken. Part of the reason for this is that many physicians are currently unaware of the value of harvesting adipose tissue for cryopreservation. Coleman’s systemization of liposuction technique [8] for the medical community increased its utility to reach this current level of use of fat grafting. It is reasonable that many physicians are not yet aware of, or comfortable enough with, cryopreservation to fully utilize the practice. Therefore, cryopreservation is, as of yet, not something that most harvesting doctors consider. As more information on its value and usefulness comes out, cryopreservation will undoubtedly become more popular. 

## Abbreviations

MSC	mesenchymal stromal cells
DMSO	dimethyl sulfoxide
PBS	phosphate buffered saline
SVF	stromal vascular fraction
CV	crystal violet
